# Cutibacterium acnes inhibits Staphylococcus lugdunensis biofilm formation through inhibition of autolysis and purine biosynthesis

**DOI:** 10.21203/rs.3.rs-8408722/v1

**Published:** 2026-01-21

**Authors:** Rayssa D. Lima, Olivia R. Bauer, Heidi Pauer, Kiana Hajiarbabi, Daniel Andrade Moreira, Thiago Estevam Parente, Rosana B. R. Ferreira

**Affiliations:** The University of Kansas; The University of Kansas; The University of Kansas; The University of Kansas; Instituto Oswaldo Cruz, Fundação Oswaldo Cruz; Instituto Oswaldo Cruz, Fundação Oswaldo Cruz; The University of Kansas

**Keywords:** Staphylococcus, Cutibacterium, skin microbiota, biofilm, extracellular DNA, autolysis, purine biosynthesis

## Abstract

*Cutibacterium acnes* is a predominant member of the human skin microbiome that plays a pivotal role in maintaining homeostasis and protecting the host against pathogen colonization. *Staphylococcus lugdunensis*, while also a resident of the skin microbiota, is an opportunistic pathogen capable of causing severe infections, associated with its ability to form biofilms. Building on our previous observation that *C. acnes* secretes molecules capable of inhibiting *S. lugdunensis* biofilm formation without inhibiting planktonic growth, we investigated the underlying molecular mechanisms of this phenomenon and its impact on pathogenicity. Here, we demonstrate that cell-free supernatants from various *C. acnes* strains exhibit dose-dependent antibiofilm activity targeting the initial stages of *S. lugdunensis* biofilm development. Additionally, extracellular molecules from *C. acnes*cultures significantly reduced the ability of *S. lugdunensis* to adhere to and invade human epithelial cells (A549) and to adhere to keratinocytes (HaCaT). Transcriptomic analysis revealed that *C. acnes*-derived molecules significantly repressed the expression of genes involved in purine biosynthesis in *S. lugdunensis*, while inducing the expression of the negative regulators of autolysis, *lrgA* and *lrgB*. Functional assays confirmed that *C. acnes*-derived molecules inhibit autolysis and extracellular DNA (eDNA) release by *S. lugdunensis*. Crucially, the addition of exogenous guanine suppressed the effect of *C. acnes* molecules on both biofilm formation and *lrgA* gene expression. Collectively, our data indicate that *C. acnes* molecules inhibit *S. lugdunensis*biofilm formation by depleting the intracellular guanine pool, which leads to repression of autolysis, thereby reducing the release of eDNA essential for biofilm structural integrity. These findings underscore the potential of exploiting interspecies microbiome interactions to better understand their role in pathogen exclusion.

## Introduction

*Staphylococcus lugdunensis* is a coagulase-negative *Staphylococcus* (CoNS) that can be found as part of the healthy human skin microbiome, localized mainly in the groin and perineum areas (1, 2). *S. lugdunensis* is also considered an important and highly virulent pathogen when compared to other CoNS species (2, 3) and is associated with a wide variety of invasive infections, including bacteremia and osteomyelitis (2, 4). Additionally, *S. lugdunensis* is known to cause very aggressive valvular endocarditis, requiring intense antimicrobial and surgical treatment, in addition to presenting high mortality rates (2). For these reasons, *S. lugdunensis* has been gaining attention in recent years.

Like other *Staphylococcus* species, the ability to form biofilms is one of *S. lugdunensis* main virulence factors, ensuring its persistence and survival in the host and the environment (5). Biofilms are formed when bacterial cells attach to a biotic, such as heart valves, or abiotic, such as a catheter, surface and produce a matrix of extracellular polymeric substances (EPS) (6, 7). The EPS protects microorganisms from the external environment and can ensure bacterial survival even under antibiotic treatment and host immune activation (6, 8). In fact, biofilms can be up to 1,000 times more resistant to antibiotics than planktonic bacteria (9). The difficulty in treating these infections leads to prolonged hospital stays and increased mortality rates (7).

In recent years, the human microbiota has been explored as a possible source of new compounds with anti-infective potential (10-14). As the largest organ in the human body, the skin is home to microorganisms that are important not only for maintaining homeostasis but also for protecting against pathogens and therefore may serve as an underexplored source of such bioactive compounds (13, 15, 16). Indeed, we and others have shown that bacteria from the skin microbiota can produce molecules with antimicrobial and antivirulence activity (10, 13, 17, 18).

*Cutibacterium acnes* is one of the most predominant bacterial species in the human skin microbiota, being found mostly in hair follicles and sebaceous glands (19, 20). Despite being associated with infections, such as periprosthetic joint infections, *C. acnes* is mostly described as a beneficial microorganism with an important role for the maintenance of skin homeostasis and protection against pathogens (20-23). Previously, we showed that *C. acnes* cell-free supernatant contains heat- and protease-resistant molecules with strong antibiofilm activity against *S. lugdunensis* (24). This activity did not affect planktonic growth, and it was specific to two *Staphylococcus* species, *S. lugdunensis* and *S. hominis*. Here, we further analyze the impact of *C. acnes*-produced molecules on *S. lugdunensis* virulence and gene expression, determining their potential mechanism of activity. We show that *C. acnes*-derived molecules repress *S. lugdunensis* biofilm formation through the repression of genes in the purine biosynthesis pathway and induction of genes that repress autolysis. This leads to the disruption of bacterial extracellular DNA (eDNA) release, a required component of the biofilm matrix of this species.

## Methodology

### Bacterial strains and growth conditions

Bacterial strains used in this study are listed in Table S1 and were stored at −80°C on trypticase soy broth (TSB; BD, Franklin Lakes, NJ, USA) supplemented with 20% glycerol (Fisher Bioreagents, Waltham, MA, USA). *S. lugdunensis* strains were grown in trypticase soy agar (TSA; BD) plates at 37°C for 24 h. *C. acnes* strains were grown in anaerobic blood agar plates (Remel, Lenexa, KS, USA) at 37°C for 72 h in an anaerobiosis chamber (85% N_2_, 10% CO_2_, 5% H_2_).

#### Preparation of C. acnes cell-free conditioned medium (CFCM)

*C. acnes* CFCM was obtained as previously described by our group (24). After growth on blood agar plates at 37°C for 72 h anaerobically, one single colony of *C. acnes* was inoculated in TSB + 0.01% cysteine (Thermo Fisher Scientific, Waltham, MA, USA) under anaerobic conditions and incubated for 72 h at 37°C in anaerobiosis. After this period, *C. acnes* cultures centrifuged at 3100 x g at 15°C for 15 min, and the supernatant was filtered [Polyethersulfone (PES) 0.22 μm membrane]. The pH of the supernatant was then adjusted to 7.0, and the solution was filtered and concentrated 20 x in sterile 0.85% saline using a Speed Vac Concentrator (Savant SPD140DDA SpeedVac concentrator; Thermo Fisher Scientific). To produce control media, the method described above was performed using only TSB + 0.01% cysteine media, without the bacterial inoculum.

### Biofilm formation assays

The biofilm formation assay was performed in 96-well, flat-bottomed polystyrene plates (TPP, Trasadingen, Switzerland), as previously described (10). Briefly, *S. lugdunensis* was grown in TSA at 37°C for 24 h and bacterial colonies were used to prepare a cell suspension (OD_600nm_ 0.1). To analyze the impact of *C. acnes* CFCM on biofilm formation, TSB + 1% glucose with either 10% (or the described concentrations) of CFCM or control media was inoculated with 10% of the *S. lugdunensis* bacterial suspension and incubated for 24 h at 37°C. After incubation, planktonic cells were removed from wells by pipetting, and the biofilm was then washed three times with phosphate buffered saline (PBS; Thermo Fisher Scientific), fixed at 60°C for 1 h, and stained with a 0.1% safranin solution (Sigma-Aldrich, St. Louis, MO, USA) at room temperature for 15 min. The wells were then washed twice with PBS, and 150 μL of 95% ethanol (Decon laboratories, King of Prussia, PA, USA) was added. The optical density (OD_492nm_) was measured after 30 min. To evaluate the effect of *C. acnes* CFCM on *S. lugdunensis* biofilm formation at different time points, 0.1% crystal violet was used instead of safranin due to the expected low biomass at these timepoints and OD_570nm_ was obtained. The effect of purine supplementation on biofilm formation in the presence of control media or CFCM was evaluated in TSB + 1% glucose supplemented with different concentrations of guanine, adenine, hypoxanthine (250 μM, 1 mM, 2 mM, and 4 mM; Sigma-Aldrich) and inosine monophosphate (IMP) (2.8 mM, 5.7 mM, 14 mM; Sigma-Aldrich). To evaluate the role of extracellular DNA on *S. lugdunensis* biofilm formation, the biofilm assay was also performed with the addition of DNase I (5 μg/mL; Roche, Indianapolis, IN, USA). Experiments were performed in triplicate in at least three independent days.

### Mature biofilm assays

To determine the role of eDNA on *S. lugdunensis* mature biofilms, biofilms were inoculated as described above, without control media or CFCM. After 24 h of incubation at 37°C, planktonic cells were removed and the biofilm was washed twice with sterile PBS. Then, a solution of DNase I (50 μg/mL) was added into the wells, and the plate was incubated at 37°C for 1 h. The plate was washed once with PBS, fixed and stained with safranin as described above, and the optical density (OD_492nm_) was measured. For the control of the experiment, PBS was used instead of a DNase I solution. Experiments were performed in triplicate in at least two independent days.

### Determination of colony forming units (CFU) of biofilms

For CFU determination, the biofilm assay was performed on 24-well, flat-bottomed polystyrene plates (TPP). After 24 h of growth at 37°C, planktonic cells were removed, the wells were washed three times with PBS, the plate was sonicated (Branson Ultrasonic 2800, Danbury, CT, USA) for 30 seconds at 40 kHz, and the wells were scraped using a sterile tip for 1 min. The content of wells was then serially diluted and inoculated in TSA. After 24 h, colonies were counted to determine CFU/mL. The experiment was performed in triplicate in two independent days.

#### Evaluation of the effect of C. acnes CFCM on S. lugdunensis planktonic growth

An *S. lugdunensis* overnight culture was diluted to an OD_600nm_ of 0.05 in TSB containing either 2X CFCM or control media in a 96-well polystyrene microtiter plate. The plate was then incubated at 37°C under shaking (250 rpm), and the OD_600nm_ was measured every hour for 24 h using a microplate reader (SpectraMAx i3 spectrophotometer; Molecular Devices, San Jose, USA). The experiment was performed in triplicate in two independent days.

### Adhesion and invasion assays

A549 cells (5 x 10^4^ cells/well ATCC, Manassas, VA, USA) and HaCaT (10^5^ cells/well; Cytion, Sioux Fall, SD, USA) cells were plated into two 24-well polystyrene plates. Plate 1 was used to obtain the total number of bacterial cells associated with host cells, and plate 2 was used to obtain the number of bacterial cells that invaded host cells. Each plate containing Dulbecco's Modified Eagle Medium (DMEM; Thermo Fisher Scientific) + 10% heat-inactivated fetal bovine serum (FBS; Cytiva, Marlborough, MA, USA) was incubated at 37°C in a CO_2_ atmosphere for 24 h (A549) or 48 h (HaCaT) (25-28). After this incubation, the media was discarded, the cells were washed with PBS, and fresh DMEM medium containing 1% heat-inactivated FBS was added 1 h prior to infection. An *S. lugdunensis* overnight culture was diluted to an OD_600nm_ 0.05 in TSB with either 10% CFCM or control media and incubated at 37°C under shaking (250 rpm) until it reached an OD_600nm_ of 0.4. Then, cultures were centrifuged (13000 x g for 5 minutes), the supernatant was discarded, and the pellet was washed with PBS (Corning Inc., Corning, NY, USA). After a new centrifugation step, the pellet was resuspended in DMEM and A549 or HaCaT cells were infected at a MOI of 100:1, and the plates were incubated at 37°C in a CO_2_ incubator for 2 h or 90 minutes, respectively. The media was discarded, and the wells from plate 1 washed twice with sterile PBS (Corning Inc.), while plate 2 was washed once. The cells in plate 1 were then lysed with 0.1% Triton X-100 (Thermo Scientific) for 10 minutes at room temperature. The content of each well was then serially diluted and inoculated onto TSA plates, which were incubated at 37°C for 24 h for determination of CFU/mL. Plate 2 was treated with gentamicin sulfate (200 μg/mL; Sigma-Aldrich) and lysostaphin (5 μg/mL; Sigma- Aldrich) for 1 h at 37°C. Then, the wells were washed twice with sterile PBS (Corning Inc.), the cells were lysed with 0.1% Triton X-100, and the content of each well serially diluted as described above for determination of CFU/mL. Adhesion was determined by subtracting the CFU/mL obtained from plate 1 from the CFU/mL obtained from plate 2. Since *S. lugdunensis* was unable to invade HaCaT cells, values for plate 1 represent adhesion to these cells. Each experiment was performed with 5 replicates in at least two independent days.

### RNA extraction

*S. lugdunensis* was grown with either 2 X CFCM or control media until it reached an OD_600nm_ of 0.4, as described for the adhesion and invasion assays. After incubation, RNA protect Bacteria Reagent (Qiagen, Germantown, MD, USA) was added, as per the manufacturer’s recommendations. The culture + RNA protect was homogenized for 5 seconds using a vortex and kept at room temperature for 5 minutes. The mixture was then centrifuged for 10 minutes (12000 x g) and the supernatant discarded. The pellets were air dried, and a lysis solution containing 20 mg/mL lysozyme (MIDSCI, Fenton, MO, USA), 40 μg/mL lysostaphin (Sigma- Aldrich), and 4 mg/mL proteinase K (Sigma-Aldrich) was added followed by an incubation step at 37°C for 1 h (homogenization was done using a vortex for 10 seconds every 10 minutes). Purification was performed using the Promega SV Total RNA Isolation System following the manufacturer’s instructions. To remove contaminating DNA, total RNA was treated with TURBO DNase (Invitrogen, Waltham, MA, USA) according to the manufacturer’s instructions, followed by an additional purification step using the SV Total RNA Isolation System (Promega), according to the manufacturer’s recommendations. RNA concentration and purity were assessed using an ND-1000 NanoDrop 246 spectrophotometer (Thermo Fisher Scientific).

### RNA sequencing and analysis

Ribosomal RNA was depleted using the NEBNext^®^ rRNA Depletion Kit (Bacteria; New England Biolabs, Ipswich, MA, USA). Then, mRNA was reverse transcribed into cDNA and sequenced with assistance from the Genome Sequencing Core at the University of Kansas using a Nextseq 2000 PI (Illumina) to produce 100-bp single-end reads with sequencing depth estimated to be about 4.4x. The data was processed and analyzed at the Bioinformatics Platform at Fiocruz using the default settings of the nf-core/rnaseq pipeline (version 3.14.0; https://nf-co.re/rnaseq/3.22.2) (29). In summary, the pipeline employs FastQC to assess read quality and Trim Galore! for the removal of adaptors and low-quality regions. The reads were then mapped to the reference genome of *S. lugdunensis* strain ATCC 43809, and differential gene expression between control media and CFCM was calculated using DESeq2, with thresholds set at a 1.5-fold change compared to control and adjusted P-value < 0.05. Metabolic pathways for each of the affected genes were determined using the EggNOG pathway database v. 6.0 (http://eggnogdb.embl.de) (30). To do this, gene IDs were searched manually in the database, and genes were categorized based on the resulting classification.

### Real Time quantitative PCR (RT-qPCR)

*S. lugdunensis* RNA was extracted as described above, after *S. lugdunensis* was maintained in the presence of control media (−/+ 2 mM guanine) or CFCM (−/+ 2 mM guanine) in TSB until reaching an OD_600nm_ of 0.4. Following DNase treatment and RNA cleanup (as described above), cDNA synthesis was performed using Go ScriptTM Reverse Transcription System (Promega, Madison, WI, USA). qRT-PCR was conducted on a QuantStudio^™^ 3 Real-Time PCR System (Applied Biosystems, Waltham, MA, USA) using PowerTrack^™^ SYBR^™^ Green Master Mix (Applied Biosystems). Gene expression levels were normalized to the housekeeping gene *aroE*, which encodes a shikimate dehydrogenase, and relative expression was calculated using the 2^−ΔΔCt^ method (31). The primers used are listed in Table S2.

### Quantification of extracellular DNA (eDNA)

eDNA from *S. lugdunensis* biofilms grown in the presence of control media or *C. acnes* CFCM was quantified using the eDNA-specific stain DiTO-1 (AAT Bioquest, Pleasanton, CA, USA) (32). Biofilms were formed for 24 h as described earlier, and the wells were washed three times with sterile PBS and stained with 1 μM DiTO for 15 minutes. The stain was removed, and the wells were washed three additional times with PBS. Then, 125 μL of PBS were added to the wells and the biofilm biomass was removed by scraping with sterile pipettes tips. The content was transferred to a flat-bottomed, black-walled 96-well plate and the fluorescence (excitation, 485/20; emission, 528/20) and the absorbance (OD_600nm_) were measured using a plate reader (Varioskan LUX, Thermo Fisher Scientific). Fluorescence values were normalized by bacterial growth (OD_600nm_). Each experiment was performed with 6 replicates in two independent days.

### Autolysis assay

Triton X-100 induced autolysis assays were performed as described by others with modifications (33). *S. lugdunensis* was incubated in the presence of either control media or CFCM using the same methodology described for the biofilm assays. Briefly, *S. lugdunensis* colonies were used to prepare a cell suspension (OD_600nm_ of 0.1), and TSB with either 10% of CFCM or control media was inoculated with 10% of this suspension and incubated for 24 h at 37°C. Then, cultures were centrifuged at 4000 x g for 12 minutes and the supernatant was discarded. The bacterial pellets were washed with cold sterile water and centrifuged at 4000 x g for 12 minutes. After that, the pellets were resuspended in a 0.05% Triton X-100 solution prepared in 0.05 M Tris-HCl buffer (pH 7.0). The suspensions were transferred to 96-well plates and incubated at 30°C with shaking at 150 rpm. The OD_600nm_ was measured every hour. Each experiment was performed with 6 replicates in two independent days and reported as percentage of autolysis over time, where the initial OD_600nm_ was the baseline of no lysis.

### Statistics

Results with comparisons between two groups were analyzed using the Student’s *t*-test, while for the analysis with more than two groups, the one-way ANOVA test was used instead (Prism 10, GraphPad). Experiments with two independent variables were analyzed using the two-way ANOVA test (Prism 10, GraphPad).

## Results

### C. acnes molecules inhibit S. lugdunensis biofilm formation at early stages in a dose dependent manner

**Figure 1:**
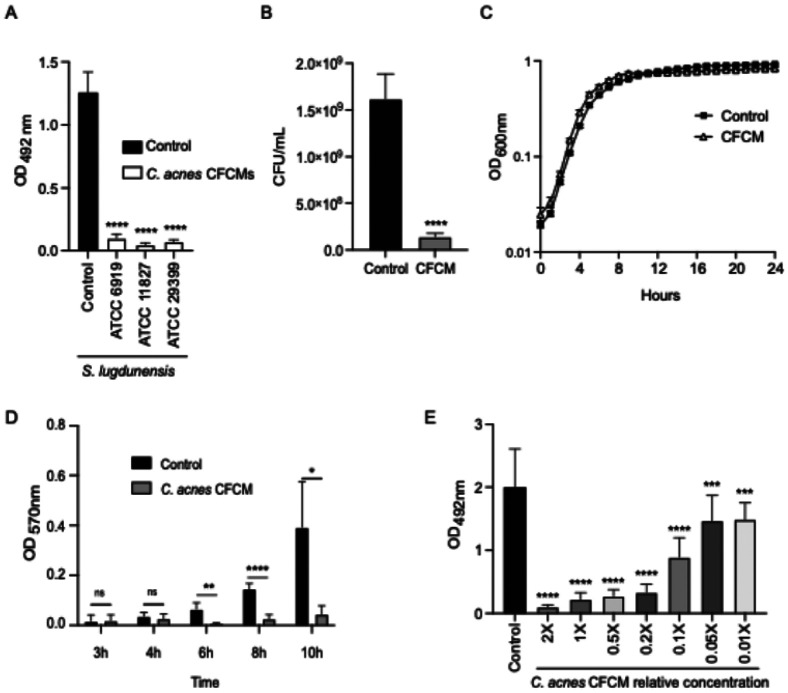
*C. acnes* cell-free conditioned media (CFCM) inhibits *S. lugdunensis* biofilm formation at early stages in a dose-dependent manner. A. Biofilm formation of *S. lugdunensis* grown in the presence of control media or CFCM from three *C. acnes* strains (ATCC 6919, ATCC 11827, and ATCC 29399). B. Cell counts of *S. lugdunensis* recovered from biofilms formed in the presence of control media or *C. acnes* CFCM (ATCC 11827). C. Growth curves of *S. lugdunensis* grown in the presence of control media or *C. acnes* CFCM (ATCC 11827). D. Biofilm formation of *S. lugdunensis*grown in the presence of control media or *C. acnes* CFCM (ATCC 11827) at different timepoints. E. Biofilm formation of *S. lugdunensis* grown in the presence of control media or with different concentrations of *C. acnes* CFCM (ATCC 11827). OD: optical density; CFU: colony-forming units; *: *P* < 0.05; **: *P* < 0.01; ***: *P* < 0.001; ****: *P* < 0.0001; ns: not significant.

To evaluate if other *C. acnes* strains produce molecules with anti-biofilm activity or whether the effect previously observed by our group is strain-specific, we tested CFCM from three different *C. acnes* ATCC strains against *S. lugdunensis* biofilm formation. All CFCMs were able to strongly inhibit *S. lugdunensis* biofilm formation, as observed by biofilm matrix staining (Fig. 1A). *C. acnes* ATCC 11827 exhibited the strongest inhibitory activity and therefore was selected for the subsequent experiments described herein. We further confirmed that the decrease in biofilm matrix staining was due to fewer bacteria present in the biofilms, as fewer bacterial colonies were recovered from biofilms formed in the presence of *C. acnes*-produced molecules compared to the control condition (Fig. 1B). Furthermore, since we are using a different *C. acnes* strain than previously reported (24), we confirmed that the molecules present on the CFCM of this strain did not affect *S. lugdunensis* planktonic growth (Fig. 1C). Additionally, we showed that the antibiofilm activity can be observed after 6 hours of *S. lugdunensis* growth in the presence of CFCM (Fig. 1D) and that this effect remains constant throughout biofilm growth. When different concentrations of *C. acnes* CFCM were used, we observed that the antibiofilm activity is dose-dependent (Fig. 1E). Altogether, these data suggest that multiple *C. acnes* strains produce molecules that strongly inhibit early stages of *S. lugdunensis* biofilm formation without affecting its planktonic growth.

### C. acnes molecules inhibit S. lugdunensis adhesion and invasion of human epithelial cells

**Figure 2:**
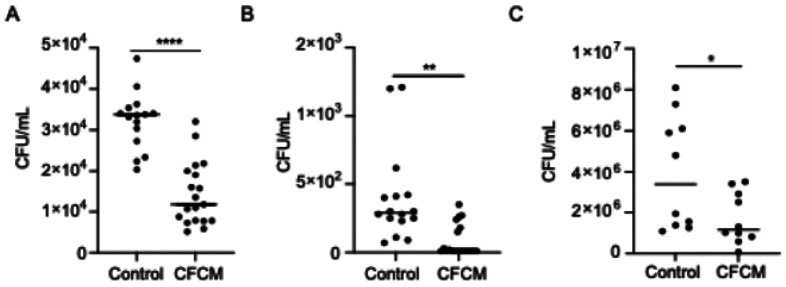
Growth of *S. lugdunensis* in the presence of *C. acnes* cell-free conditioned media (CFCM) reduces adhesion and invasion of host cells. A. Cell counts of *S. lugdunensis* recovered from intact A549 epithelial cells after growing in the presence of control media or *C. acnes* CFCM (ATCC 11827). B. Cell counts of *S. lugdunensis* internalized by A549 epithelial cells after growing in the presence of control media or *C. acnes*CFCM (ATCC 11827). C. Cell counts of *S. lugdunensis* recovered from intact HaCaT keratinocytes after growing in the presence of control media or *C. acnes* CFCM (ATCC 11827). CFU: colony-forming units; **: *P* < 0.01; ****: *P* < 0.0001.

Given the observation that the impact of *C. acnes* CFCM started during the early stages of *S. lugdunensis* biofilm formation, we also evaluated if growth in the presence of CFCM would also impact the ability of *S. lugdunensis* to adhere to or invade host cells, important steps for bacterial pathogenesis. Our data revealed that *S. lugdunensis* grown up to early stationary phase in the presence of *C. acnes* CFCM display a significantly lower ability to adhere to and invade A549 epithelial cells (Fig. 2A and B). Furthermore, *C. acnes* CFCM also decreased the ability of *S. lugdunensis* to adhere to keratinocytes (HaCaT cell line), the main cell type in the epidermis (Fig. 2C). No invasion of keratinocytes was observed under the conditions evaluated. Notably, in these assays, host cells were not in contact with *C. acnes* molecules, indicating that the effect of CFCM is on the pathogen. Our data supports the idea that *C. acnes*-derived molecules reprogram *S. lugdunensis* to a less virulent profile, affecting important steps of host colonization and pathogenesis.

### C. acnes molecules affect S. lugdunensis gene expression

**Figure 3:**
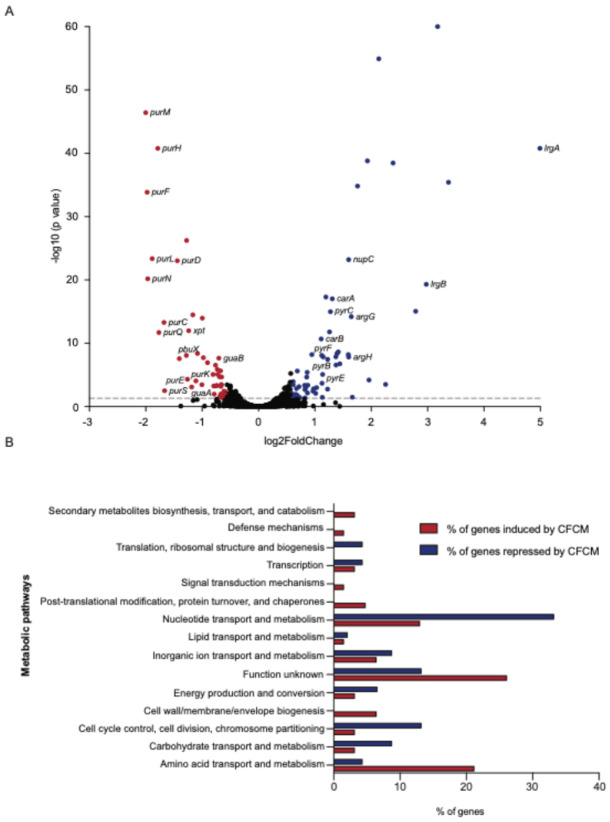
Growth in the presence of *C. acnes* cell-free conditioned media (CFCM) significantly affects the expression of *S. lugdunensis* genes associated with nucleotide and amino acid transport and metabolism. A. Volcano plot showing log_2_ fold changes and −log_10_ *P*-values induced by growth of *S. lugdunensis* in the presence of *C. acnes* CFCM (ATCC 11827) by RNA-seq. Genes significantly down- and up-regulated in *S. lugdunensis* in response to *C. acnes* CFCM relative to control media (adj. *P*<0.05) are shown in red and blue, respectively. The grey dashed line marks the 1.5-fold regulation cut-off. B. Metabolic pathways affected in *S. lugdunensis* after growth with *C. acnes* CFCM, as determined by RNA-seq and EggNOG.

Given the activity of *C. acnes* molecules on biofilm formation and interactions with host cells, we next sought to determine how *C. acnes* CFCM was affecting these *S. lugdunensis* traits. To do that, we evaluated the impact of *C. acnes* molecules on the transcriptome of *S. lugdunensis* by performing mRNA sequencing (RNA-seq) of cultures grown in the presence of *C. acnes* CFCM or control media. We detected transcripts for 2291 genes in at least one of the conditions tested (control or CFCM), representing 95.2% of the *S. lugdunensis* genome. Among them, 106 genes were differentially regulated when *S. lugdunensis* was grown in the presence of *C. acnes* CFCM compared to control media (Fig. 3A, Tables S3 and S4).

Among the 61 genes that were significantly induced by *C. acnes* CFCM, there was an overrepresentation of genes involved in amino acid transport and metabolism (Fig. 3B), which include genes in the arginine (*arcA*, *argG*, *argH*, *ureC*), aspartate (pgap_annot_002103, pgap_annot_002102), ornithine (*speC*, *potE*), and branched-chain amino acid (valine, leucine, and isoleucine) (*alsS*) pathways. Furthermore, several genes from the pyrimidine biosynthesis pathway were significantly regulated by CFCM, including *carA*, *carB*, *pyrB*, *pyrC*, *pyrE*, and *pyrF*. Besides these metabolic genes, the genes *lrgA* and *lrgB*, repressors of autolysis, were among the genes most up-regulated by CFCM (31.8-fold and 7.9-fold, respectively) (Fig. 3A).

Among the 45 genes that were significantly repressed by *C. acnes* CFCM, we observed a strong presence of genes involved in nucleotide transport and metabolism (Fig. 3B), including almost all genes in the purine *de novo* synthesis pathway (*purC*, *purS*, *purQ*, *purL*, *purF*, *purM*, *purN*, *purH*, *purD*, *guaA*, *guaB*), as well as genes involved in the purine salvage pathway (*xpt* and *pbuX*). On the other hand, the amino acid transport and metabolism pathway had a higher percentage of induced genes, apart from the ones with unknown function.

### Addition of guanine suppresses the effect of C. acnes produced molecules on biofilm formation and lrgA gene expression.

**Figure 4:**
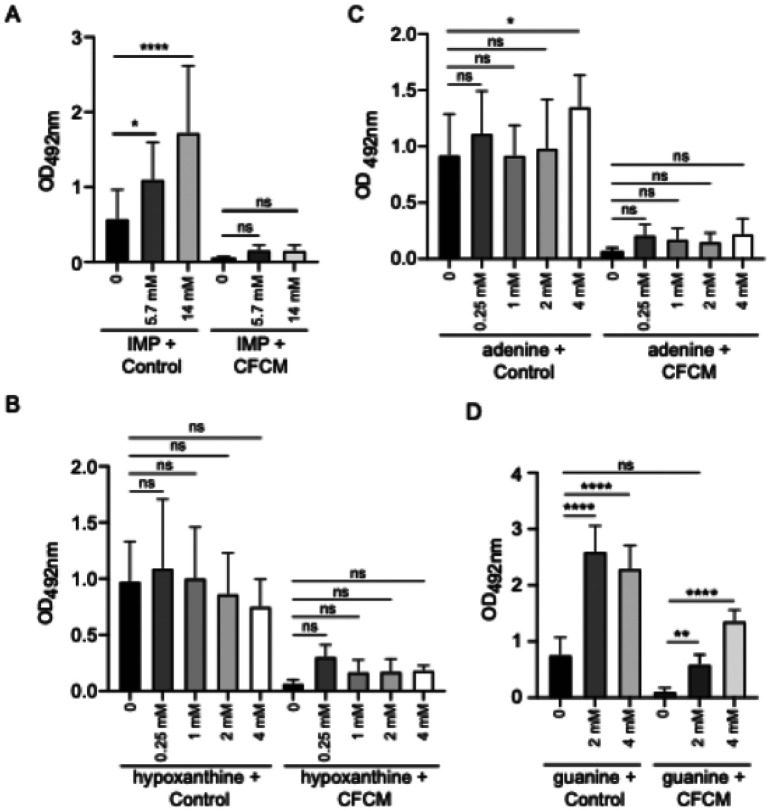
Impact of the addition of purines on the anti-biofilm activity of *C. acnes* cell-free conditioned media (CFCM) against *S. lugdunensis* biofilm formation. A. Biofilm formation of *S. lugdunensis* grown in the presence of different concentrations of inosine monophosphate (IMP; 0, 5.7, and 14 mM) with either control media or *C. acnes*CFCM (ATCC 11827). B. Biofilm formation of *S. lugdunensis* grown in the presence of different concentrations of guanine (0, 2, and 4 mM) with either control media or *C. acnes* CFCM (ATCC 11827). C. Biofilm formation of *S. lugdunensis* grown in the presence of different concentrations of adenine (0, 0.25, 1, 2, and 4 mM) with either control media or *C. acnes* CFCM (ATCC 11827). D. Biofilm formation of *S. lugdunensis* grown in the presence of different concentrations of hypoxanthine (0, 0.25, 1, 2, and 4 mM) with either control media or *C. acnes* CFCM (ATCC 11827). OD: optical density; *: *P* < 0.05; **: *P* < 0.01; ***: *P* < 0.001; ****: *P* < 0.0001; ns: not significant.

Since both biofilm formation and transcription of *S. lugdunensis* genes involved in the *de novo* pathway for purine synthesis were strongly inhibited by *C. acnes* CFCM, we hypothesized that repression of nucleotide metabolism could be the cause for the biofilm phenotype. To test this hypothesis, we added several intermediates in purine metabolism to *S. lugdunensis* cultures and tested if these treatments could rescue biofilm formation in the presence of *C. acnes* CFCM. First, we tested if the addition of inosine monophosphate (IMP) would suppress the biofilm phenotype elicited by CFCM (Fig. 4). IMP is the product of the *de novo* purine synthesis and the universal precursor for adenosine monophosphate (AMP) and guanosine monophosphate (GMP). Addition of IMP increased biofilm formation in the control condition in a dose-dependent manner but did not affect biofilm formation in the presence of *C. acnes* CFCM (Fig. 4A). Besides genes involved in *de novo* purine synthesis, the expression of some genes involved in the use of intermediates of the purine salvage pathway (*guaA*, *guaB*, *xpt*, and *pbuX*) was also inhibited by CFCM. The salvage pathway allows bacteria to recycle purine bases (adenine, guanine, and hypoxanthine) to generate nucleotides. Therefore, we next tested if addition of purine bases would suppress the CFCM biofilm phenotype. Addition of either adenine or hypoxanthine did not significantly affect *S. lugdunensis* biofilm formation in the presence of either control media or *C. acnes* CFCM, except for the addition of 4 mM of adenine, which slightly but significantly enhanced biofilm formation in the control condition (Fig. 4B and C). The addition of guanine, however, had a significant impact on biofilm formation both in the presence of control media or CFCM (Fig. 4D). The inhibitory activity of CFCM was completely abolished when guanine was added, showing that addition of this nucleotide alone can rescue biofilm formation in the presence of *C. acnes* CFCM.

**Figure 5:**
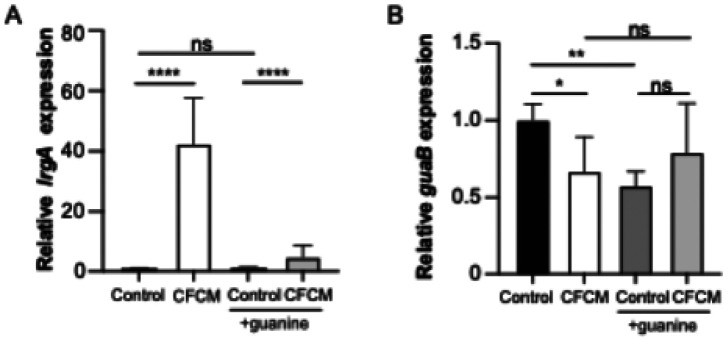
Addition of guanine suppresses the induction of *lrgA*, but not *guaA*, expression by *C. acnescell*-free conditioned media (CFCM). Relative expression of *lrgA* (A) and *guaB* (B) during *S. lugdunensis* growth in the presence of either control media or *C. acnes* CFCM (ATCC 11827), with or without guanine (2 mM). *: *P* < 0.05; **: *P* < 0.01; ****: *P* < 0.0001; ns: not significant.

We next evaluated if the addition of guanine would also suppress the effect of *C. acnes* CFCM on *S. lugdunensis* gene expression (Fig. 5). To do this, the expression of both *lrgA* and *guaA* was determined by RT-qPCR in *S. lugdunensis* cultures grown in the presence of control media or *C. acnes* CFMC, both with and without the addition of guanine. In agreement with the RNA-seq data, growth in the presence of *C. acnes* CFCM significantly induced the expression of *lrgA* and slightly repressed the expression of *guaA*. However, when guanine was added, *C. acnes* molecules failed to induce *lrgA* expression (Fig. 5A). Although addition of guanine to the control significantly repressed *guaA* expression, it did not significantly affect gene expression in the presence of CFCM (Fig. 5B). Altogether, these data indicate an important role of the *S. lugdunensis* GTP synthesis pathway and *lrgA* expression in the antibiofilm activity of *C. acnes* CFCM.

### C. acnes -produced molecules reduce S. lugdunensis autolysis and extracellular DNA release

**Figure 6:**
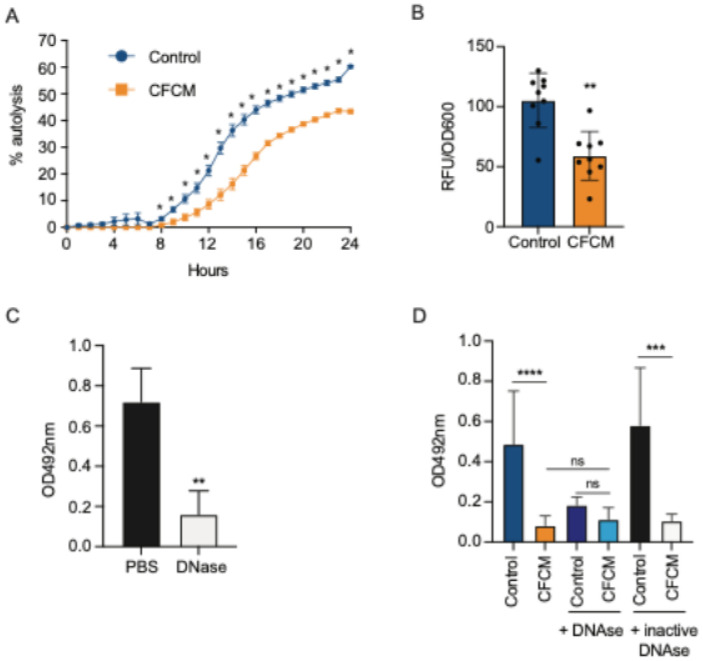
*C. acnes* cell-free conditioned media (CFCM) inhibits *S. lugdunensis* autolysis and eDNA release. A. Percentage of Triton X-100-induced autolysis of *S. lugdunensis* grown in the presence of control media or *C. acnes* CFCM (ATCC 11827). B. Fluorescence normalized by bacterial biomass (OD_600nm_) recovered from 24-hour biofilms of *S. lugdunensis* formed in the presence of control media or *C. acnes* CFCM (ATCC 11827) stained with the e-DNA-specific dye DiTO-1. C. Treatment of *S. lugdunensis* mature biofilms (24 h) with either PBS or DNase (50 μg/mL). D. Biofilm formation of *S. lugdunensis* in the presence of either control media or *C. acnes* CFCM (ATCC 11827) with active or heat-inactivated DNase (5 μg/mL). OD: optical density; *: *P* < 0.05; **: *P* < 0.01; ***: *P* < 0.001; ****: *P* < 0.0001; ns: not significant.

Since the expression of *lrgA*, an autolysis inhibitor, was highly induced in the presence of CFCM, we predicted that *C. acnes* molecules would inhibit autolysis and consequently extracellular DNA (eDNA) release. To test that, we evaluated the phenotypic consequences of *lrgA* induction by CFCM. As expected, we observed that *S. lugdunensis* grown in the presence of *C. acnes* molecules exhibited less autolysis when induced with Triton X-100, when compared to bacteria grown in the presence of control media (Fig. 6A). We then measured the amount of eDNA in biofilms treated with *C. acnes* CFCM compared to control media using an eDNA specific stain. By doing so, we observed that *S. lugdunensis* biofilms formed in the presence of CFCM produced significantly less eDNA compared to the control (Fig. 6B). This result indicates that molecules produced by *C. acnes* lead to inhibition of autolysis and, consequently, eDNA release.

eDNA is an important component of the extracellular biofilm matrix in a number of bacterial species (34, 35). Therefore, it is reasonable to expect that decreased eDNA release could lead to decreased biofilm formation. To confirm that this is the case in *S. lugdunensis*, we evaluated the role of eDNA in biofilm formation by this species. First, we treated *S. lugdunensis* pre-formed biofilms (after 24 hours of growth) with DNase, which showed a significant reduction of biofilm mass compared with treatment with PBS (vehicle) only (Fig. 6C). This data suggests that eDNA is an important component of the *S. lugdunensis* biofilm matrix. We further analyzed the role of eDNA in *S. lugdunensis* biofilm formation by adding DNase to the *S. lugdunensis* inoculum (at 0 hour) in the presence of either control or CFCM. We observed that addition of DNase prevented *S. lugdunensis* biofilm formation at a similar level as *C. acnes* CFCM (Fig. 6D). Notably, *S. lugdunensis* growth was not affected by the presence of DNAse (data not shown). The inhibition of biofilm formation was not observed when heat-inactivated DNase was added, confirming the importance of eDNA on *S. lugdunensis* biofilm formation. This confirms our hypothesis that molecules produced by *C. acnes* reduce *S. lugdunensis* biofilm formation by inhibiting autolysis, which leads to a reduction in eDNA release, which is critical for biofilm formation in this species.

## Discussion

The skin is home to millions of microorganisms that constantly interact with each other and with their host (36). It is known that these interactions can be beneficial to humans, helping to maintain skin homeostasis and protect against pathogens (37-39). *C. acnes* is one of the most abundant members of the human skin microbiota, and plays fundamental roles in this niche, such as aiding in the regulation of lipid synthesis, immune modulation, and oxidative stress mitigation (40-44). Furthermore, research has shown that *C. acnes* also helps protect the host against colonization by certain pathogens by inhibiting their growth (45, 46). Our previous work showed that *C. acnes* produces molecules with antibiofilm activity against *S. lugdunensis* (24). *S. lugdunensis* is another member of the human skin microbiota that can, under some conditions, cause severe infections, such as endocarditis. Here, we expand on this finding, exploring in more detail the inhibition of biofilm formation and other steps of bacterial virulence. Importantly, we determined the mechanism by which *C. acnes*-derived molecules inhibit biofilm formation, which involves inhibition of the purine pathway, autolysis, and the release of extracellular DNA by *S. lugdunensis*.

The impact of the purine pathway on biofilm formation has been described in *S. aureus* and other species (47-50). In these species, mutants of genes involved in the *de novo* purine biosynthesis pathway (*pur* genes) display a decreased ability to form biofilms (47-50). Furthermore, disrupting this pathway has been associated with decreased virulence in *S. aureus* (51). However, this is the first study to show that the purine biosynthesis pathway, including the purine salvage pathway that leads to GMP synthesis, is involved in the ability of *S. lugdunensis* to form biofilms, as well as adhere and invade host cells. Notably, the modulation of the expression of genes in this pathway by molecules produced by a member of the microbiome is a unique finding of our study and reveals a novel mechanism by which the microbiome can potentially interfere with pathogen colonization.

Regulation of the *pur* operon in several bacteria is known to be controlled by PurR, a transcriptional regulator that binds to the operator region of the *pur* genes, repressing their transcription (52-54). In *Bacillus subtilis*, PurR activity can be controlled by the accumulation of 5-phosphoribosyl 1-pyrophosphate (PRPP) or the end products of the purine synthesis pathway, IMP, AMP, GMP, ADP, and GDP, which block its activity, and guanosine tetraphosphate (ppGpp), which acts as an allosteric activator (52, 55, 56). It is possible that *C. acnes* CFCM contains high levels of one or more of these nucleotides, which can bind to PurR and result in the repression of *pur* genes. However, addition of IMP to *S. lugdunensis* cultures significantly increased biofilm production, indicating that increasing levels of this purine nucleotide is not enough to inhibit biofilm in the conditions used. Furthermore, addition of IMP to *C. acnes* CFCM did not increase biofilm formation compared to CFCM alone.

We also showed that *C. acnes* molecules significantly induced *lrg*AB expression. LrgA is an autolysis inhibitor, which has been shown to be important for biofilm formation in *S. aureus* (57). They modulate extracellular murein hydrolase activities, leading to decreased autolysis (58, 59). Furthermore, an *S. aureus* Δ*lrgB* strain was associated with increased cell lysis-dependent eDNA release and enhanced biofilm development *in vitro* and *in vivo* (33). In *S. aureus*, *lrgAB* transcription has been shown to be regulated by several transcriptional regulators (59-62). For example, a mutant lacking the *mgrA* gene, which encodes a MarR family winged helix-turn-helix transcriptional regulator, showed a reduced ability to form biofilms (60). This reduction was due to the induction of *lrgAB* gene expression (60, 63). In our study, transcription of *mgrA* (0.85-fold; pgap_annot_001039) was not significantly affected by the CFCM, indicating that *mgrA* does not play a role in the inhibition of biofilm by *C. acnes*. LrgAB is also known to be regulated by LytSR, a two-component regulatory system that has been shown to affect *S. aureus* biofilm development through its regulation of *lrgAB* expression (64). The LytSR system induces *lrgAB* transcription in response to a collapse in membrane potential (65, 66). Therefore, one possible mechanism by which *C. acnes* molecules are inducing *lrgAB* expression is through the LytSR system by affecting the cell membrane potential.

The connection of *lrgA* repression and purine inhibition has been observed, where an *S. aureus* mutant in *purF,* which codes for a protein involved in the first committed step in the *de novo* purine biosynthesis, was shown to produce less biofilm, less extracellular DNA levels, decreased Triton-induced cell lysis, and increased expression of *lytSR* (50). In our data, although CFCM repressed *purF* transcription, inhibiting biofilm formation, eDNA release, and triton-induced autolysis, the transcription of *lytS* and *lytR* was not affected in comparison with the control (0.85 and 0.95-fold, respectively). Nevertheless, we showed that addition of guanine, a purine predicted to be depleted by the repression of the *pur* genes, reverts both the biofilm inhibition and the induction of *lrgA* caused by *C. acnes* molecules. This data indicates an additional connection between repression of purine synthesis and induction of *lrgA* expression, independently from the transcriptional regulation of *lytSR*.

It is known that eDNA is important for biofilm formation in several other bacterial species (32, 67-69), playing an important role in adhesion to surfaces, cell-cell adhesion, biofilm dispersal, and maintaining the integrity and stability of this structure (34, 35, 70, 71). The importance of eDNA for biofilm formation in *S. lugdunensis* is still poorly understood. Some papers have shown that proteins are a major component of the biofilm matrix of this species (72, 73), while others have found that both proteins and polysaccharides contribute to the biofilm matrix in *S. lugdunensis* biofilms (74). Another study showed that the content of polysaccharides was strongly associated with biofilm mass in this species, while proteins had a weak association, and eDNA a very weak association (75). In this work, we showed for the first time that the mechanism of biofilm formation by *S. lugdunensis* is dependent on cell lysis and eDNA release, since its ability to form biofilms is impacted by *C. acnes*-produced molecules through decreased cell lysis and, consequently, decreased eDNA release.

Previously, we have shown that the bioactive molecules produced by *C. acnes*, and responsible for the effects observed in *S. lugdunensis*, are resistant to heat (100 °C for 40 min) (24). We also showed that attempting to fractionate the bioactive compounds using molecular weight filters yield only inactive fractions, suggesting that multiple molecules might be necessary for the biological activity (24). Furthermore, treatment with sodium periodate, a strong oxidizing agent that cleaves carbon-carbon bonds between adjacent carbons bearing hydroxyl groups (vicinal diols) present in some polysaccharides and dihydroxylated alkenes, completely abolished CFCM activity. This suggests a polysaccharidic nature, and efforts to definitively identify the bioactive compounds are currently underway. Nevertheless, we believe studying the combined effects of the complex mixture of compounds present in the CFCM is more biologically relevant, since this mixture is likely more similar to what *S. lugdunensis* may encounter when interacting with *C. acnes* in nature. Limitations notwhistanding, our work sheds light on potential mechanisms used by the skin microbiome to modulate important traits of other bacterial species, affecting their ability to colonize and infect hosts. This knowledge is crucial for our better understanding of how the microbiome is established and how it protects us against infections.

## Supplementary Material

This is a list of supplementary files associated with this preprint. Click to download.

• SupplementaryTables.docx

## Data Availability

The sequencing data obtained by RNA Seq in this study have been deposited in the NCBI Sequence Read Archive (SRA) under BioProject accession number XXXX.
